# Factors Influencing Climate-Smart Agriculture Practices Adoption and Crop Productivity among Smallholder Farmers in Nyimba District, Zambia

**DOI:** 10.12688/f1000research.144332.3

**Published:** 2025-03-21

**Authors:** Chavula Petros, Samuel Feyissa, Million Sileshi, Chizumba Shepande

**Affiliations:** 1 Department of Natural Resources, College of Agriculture and Environmental Sciences, Haramaya University, Dire Dawa, Dire Dawa, Ethiopia; 2 Agricultural Economics and Extension, School of Agriculture, University of Zambia, Lusaka, Lusaka Province, 10101, Zambia; 3 Agricultural Economics and Business, College of Agriculture and Environmental Sciences,, Haramaya University, Dire Dawa, Dire Dawa, Ethiopia; 4 Department of Soil Science, School of Agriculture,, University of Zambia, Lusaka, Lusaka Province, 10101, Zambia

**Keywords:** Adoption, Agriculture production, Climate-smart agriculture, Climate change, Crop productivity

## Abstract

Climate change significantly affects smallholder farmers, whose livelihoods are closely tied to the environment. This study explores factors influencing the adoption of climate-smart agriculture (CSA) practices and their impact on crop productivity among small-scale farmers in Nyimba District, Zambia. Data were collected from 194 households across 12 villages, and logistic regression and propensity score matching analyses were employed to identify key factors and evaluate CSA’s effects on crop yields.

Findings revealed that CSA adoption is influenced by factors such as education level, household size, fertilizer use, age, gender, farming experience, livestock ownership, income, farmland size, marital status, and access to climate-related information. CSA adopters experienced a 20.20% increase in overall crop yields compared to non-adopters, with a 21.50% increase in maize yields specifically.

The study underscores the need for targeted interventions to support CSA adoption through education, improved dissemination of climate information, and access to critical resources such as improved seeds and financial services. This research offers insights for policymakers and extension services to develop evidence-based strategies enhancing resilience and productivity among smallholder farmers in response to climate challenges.

## 1. Introduction

Climate change is already hindering the growth of agricultural production, both livestock and crop farming, on a global scale ( Alfani et al., 2019). Greater climate variability and shifts in climatic patterns exacerbate production risks and strain farmers’ ability to cope. These climatic changes pose threats to accessing nutritious food for urban, peri-urban, and rural communities due to decreased agricultural output and reduced household income ( Ivanova et al., 2020; Sharifi, 2021; Mossie, 2022), as well as increased risks that disrupt food markets. The 2018 assessment from the Intergovernmental Panel on Climate Change (IPCC) states that food output is impacted by climate change in most parts of the world, with negative consequences predominating over positive ones. Developing nations are particularly vulnerable to additional negative effects. In many parts of the world, there has already been and is predicted to be an acceleration of increases in the frequency and intensity of severe events, such as drought, flooding, heavy rainfall, and high maximum temperatures ( Murray & Ebi, 2012; IPCC, 2018). It is anticipated that average and seasonal maximum temperatures will keep rising along with an overall increase in average rainfall. But the distribution of these effects won’t be uniform. By the end of the twenty-first century, there will probably be more drought and scarcity of water in existing arid areas.

Climate change is projected to contribute to and/or is already causing a global reduction in cereal yields, such as maize and wheat declining by 3.8% and 5.5% respectively ( Branca et al., 2016; Ngoma et al., 2021). Several researchers warn that crop productivity will experience steep declines when temperatures exceed critical physiological thresholds for these crops ( Kaczan et al., 2013). Smallholder farmers, including poor producers, the landless, and marginalized ethnic groups, are among the most vulnerable to the impacts of climate change ( CIAT & World Bank, 2017; Makate, 2019). Their livelihoods and food security are threatened by the reduced agricultural yields and disruptions to food systems caused by climate change ( Chavula, 2022). Climate change-induced extreme weather events and shocks can have long-lasting impacts by altering investment incentives, increasing the likelihood of low-risk, low-return ventures, and decreasing the chances of successful agricultural advancements ( Branca et al., 2016; Ngoma et al., 2021). According to studies, the average yields of Zambia’s major crops, such as wheat, sorghum, and maize, are likely to be significantly affected by climate change, as the agronomic conditions for these crops may deteriorate across a significant portion of the country ( Molieleng et al., 2021; Chavula, 2022; Stahlbaumer et al., 2022).

Climate change-induced extreme events and shocks, such as droughts and floods, have a significant impact on crop production in Zambia and other Sub-Saharan African countries ( Arslan et al., 2014). However, due to the intricate nature of agricultural systems in Sub-Saharan Africa and their interrelation with the socio-economic aspects of smallholder farmers’ households, an integrated approach has been promoted to maximize productivity in the smallholder agricultural landscape and adapt to climate change. These approaches and interventions are termed ‘climate-smart agriculture (CSA)’ ( Makate, 2019; Odubote & Ajayi, 2020; Zakaria et al., 2020; Molieleng et al., 2021).

The integrated approach recognizes the complexity of agricultural systems in Sub-Saharan Africa and their interconnectedness with the socio-economic factors affecting smallholder farmers’ households ( Beedy et al., 2010). Due to adopting climate-smart agricultural practices, smallholder farmers can enhance productivity while simultaneously adapting to the impacts of climate change on their agricultural landscapes.

In Zambia, as farmers grappled with mounting economic pressures, environmental degradation, and climatic adversities towards the close of the 20th century, they turned to climate-smart agriculture (CSA) as a viable solution ( Branca et al., 2016; Ngoma et al., 2021). The initial thrust of CSA practices was to enable smallholder farmers to sustain viable production levels, thereby securing their role as active participants in the agricultural landscape. The emergence of CSA was driven by the need to mitigate the deleterious impacts of climate change on smallholder farming communities. To bolster household resilience against climatic variability and rehabilitate degraded lands, the Zambian government has been spearheading efforts to promote the widespread adoption of CSA ( Branca et al., 2016; Ngoma et al., 2021). These endeavours have been facilitated through collaborations with regional, national, and global research and development institutions ( Ngoma et al., 2021). CSA encompasses a suite of practices, including conservation agriculture, integrated pest management, organic farming methodologies, sustainable agricultural techniques, integrated nutrient management, multi-cropping systems, and agroforestry approaches. The overarching objectives of these practices are to augment household incomes, enhance agricultural productivity, and cultivate climate resilience through judicious fertilizer application and sustainable land stewardship ( Newell et al., 2019).

Given the critical role of climate-smart agriculture (CSA) in sustainable food production, the Zambian government has prioritized the promotion of climate-smart farming practices among smallholder farmers ( Arslan et al., 2014). These practices encompass organic farming, integrated pest management, agroforestry, conservation agriculture, and integrated agricultural systems, which have become fundamental components of extension and rural advisory service delivery. While previous empirical studies have explored various aspects of agricultural development in Zambia, this research specifically examines the determinants of CSA adoption and its subsequent impact on crop productivity among smallholder farmers in Nyimba district. This study contributes to the existing literature by providing a comprehensive analysis of the factors influencing CSA adoption decisions and their corresponding effects on crop yields in the context of smallholder farming systems. Understanding these determinants is crucial for policymakers and agricultural extension services to develop targeted interventions that facilitate the widespread adoption of CSA practices. Such knowledge can inform evidence-based policies and programs aimed at enhancing the resilience and productivity of smallholder farming systems in the face of climate change challenges.

### 1.1 Conceptual framework

**Figure 1.**
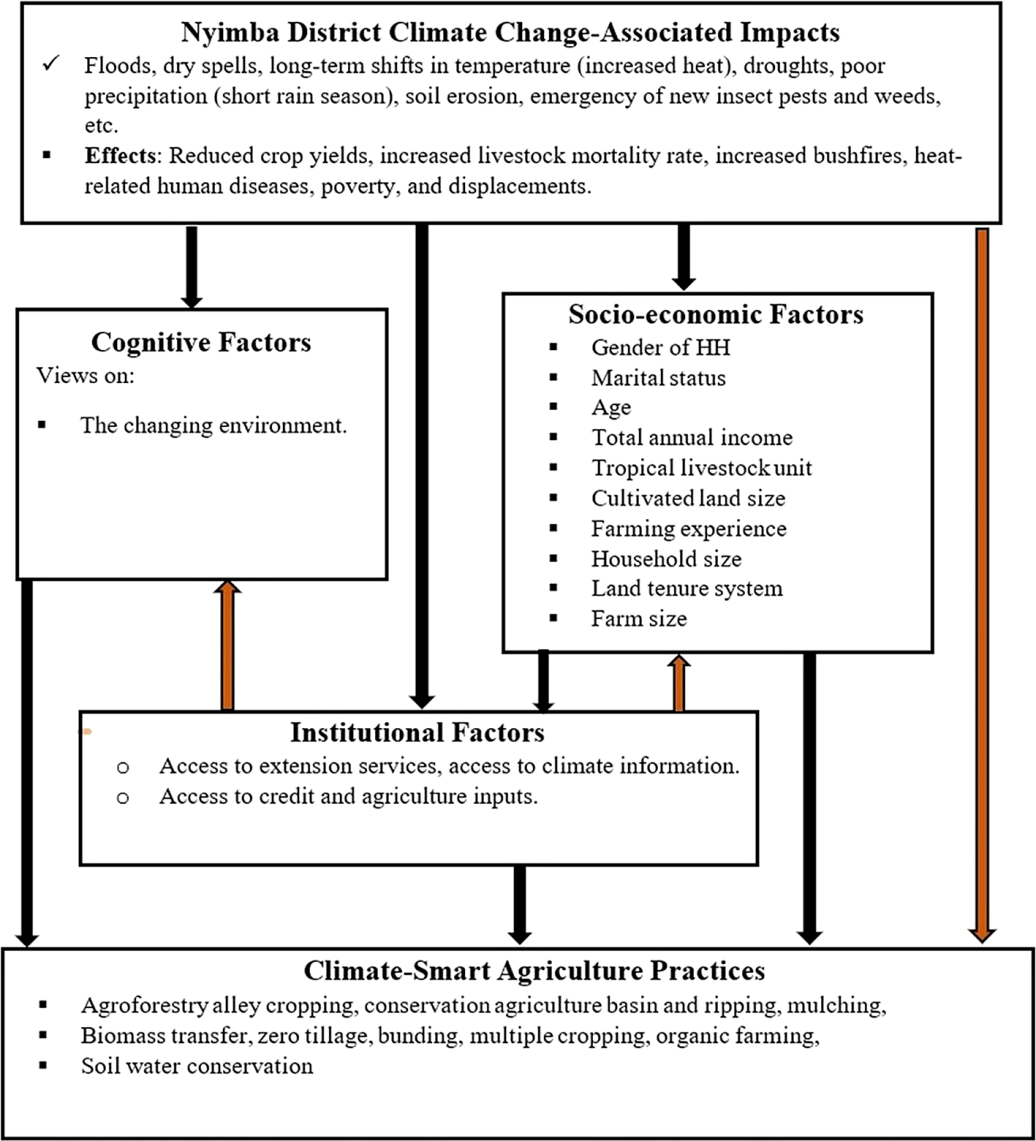
Conceptual framework based on adoption.

In the face of changing climatic conditions and climate change, climate-smart agriculture (CSA) is an approach to transform and reorient agricultural systems to ensure food security ( Chavula, 2021). Climate change disrupts food markets, putting food supply and production at risk. Strengthening farmers’ adaptive capacity and enhancing the mitigation potential and efficiency of agricultural production systems can mitigate these risks. Smallholder farmers who are aware of climate change or perceive it as a reality are more likely to adopt climate-smart agricultural practices. These farmers aim to achieve the three pillars of CSA: improving household income and productivity, enhancing resilience and adaptation, and reducing greenhouse gas emissions. The adoption of climate-smart agriculture to attain these principles is influenced by institutional, cognitive, and socioeconomic factors ( Figure 1).

## 2. Methods

### 2.1 Study area description

**2.1.1 Location**

**Figure 2.**
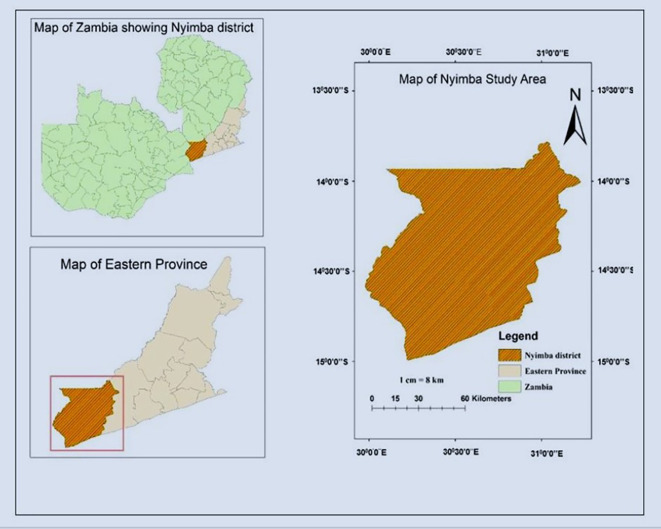
Map of study area.

The research was carried out in Nyimba district of Eastern Province Zambia. The district is situated 334 kilometers East of Lusaka Zambia’s national capital. In the South the district borders with Mozambique, North with Muchinga province, West with Lusaka province, and East with Petauke district. The district lies between latitude (13°30′1019″ and 14°55′81426″ South) and longitude (30° 48′5047″ and 31°48′20252′′East) ( Figure 2).

**2.1.2 Climate, soil and topography**

**Figure 3.**
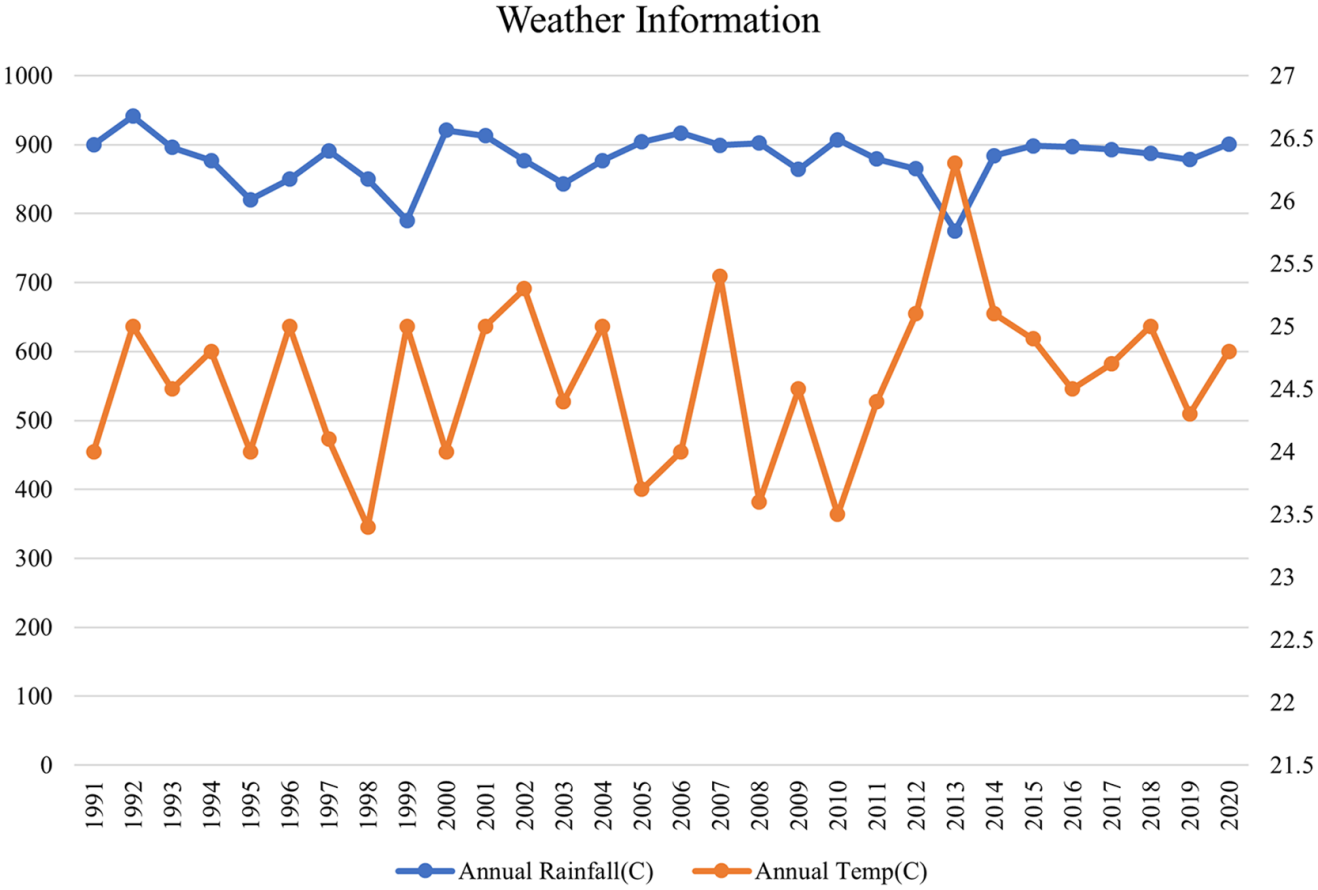
Mean annual rainfall and temperature for the study area.

Zambia is divided into three agro-ecological zones: Zone I, Zone II (subdivided into IIa and IIb), and Zone III. Nyimba district falls within Zone I, which covers the Southern and Eastern rift valleys of the Zambezi and Luangwa River basins. This zone also extends to parts of the Western and Southern provinces in the south of Zambia. The average annual rainfall in Nyimba district ranges from 600 to 900 mm, with the wettest months being December to February and a distinct dry season from May to November. The annual mean temperature is 24.2°C, while the daily temperature range is from 10.3°C to 36.5°C ( Figure 3). Topographically, the district comprises hills and plateaus, with soils characterized as Lithosol-Cambisols, while in the valleys, the soils are classified as Fluvisol-Vertisols. The elevation varies from 450-1000 m at the bottom of the Luangwa River valley, extending to the plateau near the Nyimba district center and reaching even higher altitudes on the mountain tops in the western part of the district.

**2.1.3 Vegetation type**

The Miombo woodland is the most dominant formation and habitat type in Southern Africa ( Gumbo and Dumas-Johansen, 2021; Montfort et al., 2021). Miombo woodland is also the major forest type in Zambia itself, covering approximately 45% of the entire land surface ( Kalinda, 2008). Nyimba is located in the middle of the Miombo Ecoregion, a biome with a variety of flora types that is dominated by tree species from the Caesalpinioiae subfamily of leguminous plants ( Timberlake et al., 2010). Depending on the climate, soil, landscape position, and degree of disturbance, the ecoregion’s vegetation varies in composition and structure ( Timberlake et al., 2010; Halperin et al., 2016). Nyimba is located in the arid ecozone and is characterised by four types of plants: Dry miombo woodland (i.e., *Brachystegia spiciformis, Brachystegia boehmii* and *Julbernardia globiflora*), Mopane woodland ( *i.e., Colophospermum mopan*e), Munga woodland ( *i.e., Vechellia sp.*, *Senegalia sp.*, *Combretum sp.*, and trees associated with the Papilionoideae subfamily) and Riparian Forest ( *i.e.,* mixed tree species).

**2.1.4 Land use and farming systems**

Nyimba district’s total land area is about 10,500 square kilometres according to the population and housing census of 2010. Therefore, 82% of the district population is agrarian with average household income. These households are farmers who are into mixed agriculture practices dominating the agricultural scene in the district. However, local smallholder farmers in the district practice some sort of shifting cultivation. Under this agricultural system, crops are grown in mounds and/or ridges in most cases maize. The major crops grown in include banana ( *Musa sp.*), maize ( *Zea mays*), finger millet ( *Eleusine coracana*), groundnuts ( *Arachis hypogaea*), haricot bean *(Phaseolus vulgaris)*, cowpeas ( *Vigna unguiculata* spp.) and soybean ( *Glycine max*). Multiple cropping systems are common among farming households where cultivated land is on gently and moderately steep slopes. The topography of the land in the district makes the agricultural or cultivation pattern different from other areas. Therein, the cropping system is alongside livestock production such as cattle, goats, chickens, ducks and doves. Besides agricultural activities, farmers are engaged in charcoal production, timber, firewood supply and non-timber forest products (NTFPs) from the miombo woodland for household economic gain ( *Policy Brief*, 2016).

### 2.2 Site selection

Before commencing data collection, an exploratory study was conducted to gather key information about the study area. This included data on the distances between villages, the number of farming households in each village, contact information for lead farmers, households practicing climate-smart agriculture (CSA), the locations of croplands, and central meeting points for focus group discussions (FGDs).

**2.2.1 Sampling technique**

This study utilized a multi-stage random sampling approach to recruit participants from smallholder farming communities in Zambia. These farming communities, known as agricultural camps, are designated by the Ministry of Agriculture of the Republic of Zambia. The camps group smallholder farmers’ residences within a district, facilitating easy access to agricultural extension services. From the eight agricultural camps in Nyimba District, four camps – Ndake, Central Camp, Lwende, and Ofumaya – were randomly selected for the study. These four camps collectively house 10,700 farmers. To determine an appropriate sample size, the study employed Slovin’s formula. Additionally, three villages from the four selected camps were randomly chosen for data collection. These villages were Sikwenda, Sichipale, Mawanda, Elina, Katumbila, Sichalika, Malalo, Mwenecisango, Mulivi, Lengwe, Mofu, and Yona. With a margin of error set at 0.05, the initial calculated sample size was 386 participants. However, to optimize resources, the researchers increased the margin of error to 0.1, resulting in a smaller sample size of 99 participants. Achieving this reduced sample size required additional time and financial investments, but it was a trade-off deemed necessary for the successful completion of the study.

Sample size formula: Slovin’s (1960) formula.

$$n=\frac{N}{1+Ne^{2}}$$

$$\;n=10700/(1+10700\left(0.1^{2}\right)$$

$$\;n=\frac{10700}{27.75}\;n=99.07$$

Ultimately, the researchers opted for a compromise, settling on a sample size of 194 participants, which fell between the initial calculations of 99 (with a 0.1 margin of error) and 386 (with a 0.05 margin of error). With the assistance of agricultural camp officers, farmer registers from each selected village were utilized to randomly identify potential participants using an Excel spreadsheet.

**2.2.2 Focus group discussion**

Focused group discussions (FGDs) were used to gather in-depth information on factors influencing the adoption of climate-smart agricultural practices, crop productivity, perceptions of these practices, the use of CSAPs, and views on climate change. An open-ended FGD guide was developed for this purpose. FGDs were chosen over one-on-one interviews, as they often reveal sensitive topics that individual farmers might find difficult to express fully in private interviews. Four FGDs were conducted in the study area, engaging village headmen, women, men, and youth. These discussions were held at locations convenient for participants, facilitating broad attendance. The FGDs served primarily to supplement data collected from household questionnaires.

**2.3.3 Household interviews**

The household survey questionnaire included both closed-ended and open-ended questions. The questionnaire was pre-tested six times to ensure clarity, relevance, and logical flow before conducting the household interviews. Based on the pre-test results, adjustments were made to improve its suitability. The pre-testing was conducted with smallholder farmers who were not participants in the main study. Data collection was carried out by three trained enumerators, supervised by the principal researcher, a seasoned professional in the field. The enumerators carefully reviewed and corrected the collected data at the end of each day to ensure accuracy and consistency. Subsequently, the validated information was securely backed up on the CSPRO Cloud platform ( https://www.csprousers.org/help/CSDeploy/deployment_options.html).

### 2.4 Variables specification

**2.4.1 Outcome variables**

The outcome variable for this study is the impact of adopting climate-smart agriculture practices on crop productivity among smallholder farmers’ households in Nyimba district.

**2.4.2 Dependent variables**

**2.4.2.1 Smallholder farmers’ household decision to adopt CSAPs**

The dependent variable in the study was the adoption of Climate-Smart Agricultural Practices (CSAPs) by a smallholder farmer’s household. This variable was coded as 1 if the household adopted CSAPs and 0 if it did not.

### 2.5 Propensity score matching

To evaluate the impact of Climate-Smart Agricultural Practices (CSAPs) on crop productivity, this study employed the Propensity Score Matching (PSM) method, comparing adopters and non-adopters. PSM is a statistical technique that adjusts for confounding variables across a sample population, enabling more accurate estimation of treatment effects. As outlined by Caliendo and Kopeinig (2008), the implementation of PSM involves several key steps: first, estimating propensity scores using a binary model; second, selecting an appropriate matching algorithm; third, verifying the common support condition; and fourth, assessing the quality of the matching between the treatment and control groups. The following are the steps involved in PSM:

**Step 1:** Model Specification

The logistic model was chosen for this study due to the robustness of its parameter estimations, which stems from the assumption that the error term in the equation follows a logistic distribution ( Baker and Melino, 2000; Ravallion, 2007). Therefore, the Logit model was used to estimate the probability of smallholder farmers’ adoption of CSAPs allotted to socio-economic, agro-ecological and institutional characteristics. Therein, a dependent variable considered a value of 1 for CSAPs adoption and 0 for non-CSAPs adopters.

$$P_{i}=P\left(Y=1|X\right)\;$$ (1)

In line with Pindyck and Rubinfeld (1981), the cumulative logistic probability function is specified as follows;

$$P_{i}=F\left(Z_{i}\right)=F[a+\sum_{i=1}^{m}\beta_{i}X_{i}]=\left[\frac{1}{1+e^{-(a+\sum \beta_{i}X_{i})}}\right]\;$$ (2)

where *e* represents the base of natural logs, *X _i_* represents the *i* ^th^ explanatory variable, *P*_*i*_ the probability that a household adopted CSAPs, *α* and *β _i_* are parameters to be estimated.

Expressing the logistic model in terms of odds and log-odds aids in interpreting the coefficients ( Gujarati, 1995). The odds ratio quantifies the relative probability of an individual participating ( *P*_i_) versus not participating (1- *P*_i_). The probability of non-participation can be calculated as:

$$\left(1-P_{i}\right)=\frac{1}{1+e^{\mathrm{zi}}}\;$$ (3)

$$\left(\frac{P_{i}}{1+P_{i}}\right)=\left[\frac{1+e^{\mathrm{zi}}}{1+e^{-\mathrm{zi}}}\right]=e^{\mathrm{zi}}\;$$ (4)

Alternatively,

$$\left(\frac{P_{i}}{1+P_{i}}\right)=\left[\frac{1+e^{\mathrm{zi}}}{1+e^{-\mathrm{zi}}}\right]=e^{\left[a+\sum B_{i}X_{i}\right]}\;$$ (5)

Taking the natural logarithms of equation (3.5) will give the logit model as indicated below.

$$Z_{i}=\ln\left(\frac{P_{i}}{1-P_{i}}\right)=a+B_{1}X_{1i}+B_{2}X_{2i}+\ldots B_{m}X_{\mathrm{mi}}$$ (6)

If consider a disturbance term, *μ*_i_, the logit model becomes

$$Z_{i}=a+\sum_{t=1}^{m}B_{t}X_{\mathrm{ti}}+\mu_{i}$$

So, the binary logit will become:

Pr(pp)=f(X) (7)

Where *pp* is CSAPs adoption, *f*( *X*) is the dependent variable project participation and *X* is a vector of observable covariates of the households. The dependent variable will take a value of 1 for CSAPs adoption and 0 for non-adopters.

In addition to the estimated coefficients, the marginal effects of the change in the explanatory variables on the probability of CSAPs adoption are also estimated. The interpretation of these marginal values will be dependent on the unit of the measurement for explanatory variables.

When an explanatory variable is binary, the marginal effects provide a reasonable estimate of the change in the likelihood of the outcome variable (Y = 1) evaluated at representative values of the regressors, such as their means.

**Step 2**: Identifying the Common Support Region and Conducting Balancing Tests

The region where the propensity score distributions of the treatment and comparison groups overlap is known as the area of common support. This needs to be identified. However, if there is a systematic difference in the observed characteristics between the dropped non-adopters of CSAPs and the retained non-adopters, sampling bias may still occur. These differences should be closely monitored to aid in the interpretation of the treatment effect. Furthermore, balancing tests can be conducted to check if the mean of the covariates (X) and the average propensity score are equal within each quantile of the propensity score distribution.

For PSM to be effective, the treatment and comparison groups must be well-balanced based on their observed covariates (X), as similar propensity scores are derived from similar observed characteristics. Achieving balance necessitates that the covariate distributions of the treated and untreated units are indistinguishable. Formally, this requires verifying that the conditional distributions P(X|T=1) and P(X|T=0) are equivalent. Here’s a version with reduced similarity:

**Step 3:** Matching adopters to non-adopters

Selecting an available data matching algorithm is the third stage. Selecting control subjects who are matched to treated subjects based on context factors that the investigator feels should be tracked is a standard process known as matching. A different one might use comparable criteria. according to propensity score, divide adopters against non-adopters. Kernel-based matching (KBM), radius matching (RM), and closest neighbour matching (NN) are the most often used matching algorithms.

**Step 4:** Matching quality

Matching quality tests could be conducted in the fourth step. Whether or whether the matching method can balance the distribution of different variables is determined by checking for matching, irrespective of quality. If there are discrepancies, it could be a sign of insufficient matching, and corrective action is advised ( Caliendo & Kopeinig, 2008). The next action is to determine if the treatment caused a difference in the impact indicators.

The difference between the mean outcome of matched adopters and nonadopters with common support conditional at the propensity score provides the average treatment effect at the treated (ATT).

**Step 5:** Sensitivity analysis

Lastly, to verify the strength of the conditional independence assumption, a sensitivity analysis will be performed. Sensitivity analysis will also be used to examine whether the influence of an unmeasured variable on the decision-making process is significant enough to compromise the matching strategy ( Ali & Abdulai, 2010). The sensitivity analysis (r-bounds test) will be performed using the Rosenbaum bound sensitivity test.

### 2.6 Ethical clearance

The study was conducted by the researcher and two supervisors, adhering to principles of integrity, objectivity, openness, respect for research participants, respect for intellectual property, confidentiality, informed consent, fidelity, and honesty. The researcher and supervisors take full responsibility for their actions and publications, ensuring that all agreements are intended to be upheld. The study was approved by the University of Zambia Directorate of Research and Graduate Studies (NASREC) on March 18, 2024, and is registered under NASREC IRB No. 00005465 (IORG No. 0005376). The principal researcher provided a written consent form for research participants to participate in the household survey, conforming to the research ethics guidelines of the University of Zambia and Haramaya University.

## 3. Results and discussion

### 3.1 Effect of climate-smart practices on crop productivity among smallholder farmers, in Nyimba, Zambia

**3.1.1 Characteristics of the participant smallholder farmers**

The household survey involved 194 randomly selected smallholder farmers from the study area. These farmers were interviewed regarding their crop production and use of various Climate-Smart Agriculture (CSA) practices. The study presents the survey findings, beginning with participants’ demographic characteristics ( Table 1), and continues with sections on crop production and productivity, CSA practice adoption, constraints to CSA adoption, the effects of CSA on crop productivity, and factors influencing productivity. In total, 339 field plots of various crops were surveyed from the 194 farmer participants. Table 2 provides comprehensive demographic and socio-economic information about the respondents. The mean age of respondents was 46 years (standard deviation: 14.59), with the majority of households (62.18%) being male-headed. Most participants (69.43%) were married. Respondents had an average of 5.49 years of formal education (standard deviation: 3.5) and 26.22 years of farming experience (standard deviation: 15.55). They had resided in the area for an average of 30.92 years (standard deviation: 18.68). The mean household size was 5.42 (standard deviation: 2.14), and the average total annual income was K5,472.68 (USD 331.68 at an exchange rate of K16.5 per USD), with a standard deviation of 7,626.52. Additionally, 57.51% of respondents participated in off-farm activities. In terms of agricultural practices, 78.76% of respondents used improved seed varieties, and the mean farm size was 3.396 hectares (standard deviation: 3.363), with all land under customary tenure. The average cultivated land was 1.83 hectares (standard deviation: 1.45). Smallholder farmers grew, on average, two different crops (standard deviation: 0.930).

**Table 1.** Independent variables of the study.

Variable name	Description	Measurement	Expected sign
**Continuous variables**			
Age	Years of household head	Continuous	**+**
Household size	Household number of people	Continuous	**+**
Income	Household average income (ZMK)	Continuous	**+**
Fertilizer	Amount of fertilizer applied (kg)	Continuous	**+**
Education	Number of years in school	Continuous	**+**
Farmland size	Size of farmland	Continuous	**-**
Experience	Household head farming experience	Continuous	**+**
TLU	Tropical livestock unit	Continuous	**+**
**Dummy variables**			
Sex	Gender of household head (1=Male, 0=Female)	Dummy	**+**
Information	Access to climate information (1=Yes, 0=Otherwise)	Dummy	**-**
Marital status	Household head if married (1=Yes, 0=Otherwise	Dummy	**+**
Credit	Household access to credit (1=Yes, 0=Otherwise)	Dummy	**+**
Extension	Access to extension services (Yes=1, 0=Otherwise)	Dummy	**-**

**Table 2.** Independent variables of the study.

Variable name	Description	Mean	St. Dev	%
**Continuous variables**				
Age	Years of household head	46.18	14.59	**-**
Household size	Household number of people	5.42	2.14	**-**
Income	Household average income (ZMK)	5472.69	7626.52	**-**
Living	Years of living in an area	31.00	18.68	**-**
Education	Number of years in school	5.49	3.50	**-**
Farmland size	Size of farmland	3.40	3.36	**-**
Experience	Household head farming experience	26.22	15.55	**-**
Cultivated land	Cultivated land in hectares (2021/2022)	1.83	1.45	**-**
Crops	Number of crops planted (2021/2022)	2	0.93	**-**
**Dummy variables**				
Sex	Gender of household head (1=Male, 0=Female)	-	**-**	62.18%
Information	Access to climate information (1=Yes, 0=Otherwise)	-	**-**	23.14 %
Marital status	Household head if married (1= Yes, 0=Otherwise	-	**-**	69.43%
Credit	Household access to credit (1=Yes, 0=Otherwise)	-	**-**	26.71%
Extension	Access to extension services (Yes=1, 0=Otherwise)	-	**-**	1.8%
Off-farm	Participation in off-farm activities (Yes=1, 0=Otherwise)	-	**-**	57.51%
Improved seed	Adoption of improved maize variety (Yes=1, 0=Otherwise)	-	**-**	78.76%
Land tenure	Land tenure system (1=Customary, 0=State)	-	**-**	100%

**3.1.2 Crops grown by smallholder farmers**

Regarding the crops that the farmers grew, the study discovered that the most common crop was maize, which was recorded in 194 crop plots. Groundnuts were reported in 99 plots, sunflower in 69 plots, and soybeans in 16 plots ( Table 2). It was said that the other crops—cowpea, sweet potatoes, millet, cotton, and bambara nuts—were produced in small plots.

**3.1.3 Climate-smart agriculture practices adopted by smallholder farmers**

The study’s findings shed light on the adoption of various conservation agriculture techniques across the surveyed field plots. Among these practices, pot-holing (basin) emerged as the most widely implemented method, with 61 plots (17.99%) employing this technique. Closely following was multi-cropping, which was practiced on 50 plots, accounting for 14.75% of the total. Minimum tillage, a soil conservation approach, was utilized on 34 plots, representing 10.03% of the survey sample. The ripping technique, which involves creating furrows in the soil, was observed on 32 plots (9.44%). Furthermore, 18 plots (5.31%) incorporated crop rotation as a means of maintaining soil fertility and controlling pests and diseases. The application of manure, a natural fertilizer, was recorded on 11 plots (3.24%), while alley cropping, a system that combines crops with trees or shrubs, was adopted on 9 plots (2.65%) ( Table 3). It is noteworthy that the remaining conservation agriculture techniques were tested on fewer than 10 plots each, indicating their relatively limited implementation within the surveyed area.

**Table 3.** Crops grown by smallholder farmers.

Crops Grown	Frequency	Percent	Cumulative
Maize	194	50.13	50.13
Soybeans	16	4.13	54.26
Groundnuts	99	25.58	79.84
Cowpea	2	0.52	80.36
Bambara nuts	2	0.52	80.88
Sunflower	69	17.83	98.71
Cotton	1	0.26	98.97
Sweet potatoes	3	0.78	99.74
Millet	1	0.26	100
**Total**	**387**	**100**	

**3.1.4 Number of climate smart agriculture practices adopted by smallholder farmers**

The study’s findings, as presented in Table 4, shed light on the extent of conservation agriculture (CSA) practice adoption among the surveyed plots. Notably, a substantial number of plots, 167 (49.26%), did not incorporate any CSA techniques whatsoever. This highlights a significant gap in the implementation of sustainable agricultural practices within the surveyed area. On the other hand, a considerable portion of the plots, 123 (36.28%), had adopted at least one CSA practice, indicating a positive step towards embracing more environmentally friendly farming methods. Additionally, 4 plots (12.68%) had implemented two different CSA practices simultaneously, demonstrating a more comprehensive approach to sustainable agriculture. Interestingly, the data revealed that a smaller number of plots had adopted multiple CSA techniques concurrently. Specifically, four plots had incorporated three distinct CSA practices, while one plot had implemented an impressive four different CSA practices. Furthermore, another plot stood out by adopting a remarkable five separate CSA techniques. Despite these instances of multiple CSA practice adoption, the overall findings suggest that the majority of farmers within the surveyed area were either not implementing any CSA techniques or had adopted only a single practice. This observation underscores the potential for further education and awareness-raising efforts to encourage the widespread adoption of multiple sustainable agricultural practices, ultimately contributing to improved soil health, crop productivity, and environmental conservation.

**Table 4.** Climate-smart agriculture practices adopted by smallholder farmers.

CSA Practices	Frequency	Percent
Ripping	32	9.44
Basin	61	17.99
Crop rotation	18	5.31
Crop residue	2	0.59
Alley cropping	9	2.65
Multi cropping	50	14.75
Contour ploughing	6	1.77
Compost	5	1.47
Manure field	11	3.24
Zero tillage	34	10.03
Bunding	2	0.59

**3.1.5 Quantities harvested for various crops (kg)**

The survey data presented in Tables 5 and 6 offers valuable insights into the crop cultivation patterns and yield outcomes among the surveyed farming community. Notably, the crops that emerged as the most prevalent choices among farmers were soybeans, maize (corn), groundnuts, and sunflowers. Across all crop types, the average harvest weight recorded was 1223.51 kg, accompanied by a substantial standard deviation of 1442.82. This variation in yields highlights the diverse factors that can influence agricultural productivity, including soil conditions, farming practices, environmental variables, and access to resources. When examining the individual crop yields, maize stood out as a prominent crop, with an impressive average harvest weight of 1766.57 kg. Therefore, the high standard deviation of 1594.23 suggests significant variations in maize yields among farmers, potentially attributable to differences in cultivation techniques, seed quality, or localized environmental conditions. Groundnuts, a staple crop in the region, exhibited an average harvest weight of 511.08 kg, with a standard deviation of 605.07. This relatively lower yield, coupled with the substantial variation, may indicate challenges faced by farmers in optimizing groundnut production, such as pest or disease pressures, or limitations in access to appropriate inputs and knowledge. Sunflowers, a valuable oilseed crop, yielded an average harvest weight of 609.67 kg, with a standard deviation of 513.02. While the average yield appears moderate, the substantial variation observed could be attributed to factors like soil fertility, water availability, or sunflower variety selection. Notably, soybeans emerged as a crop with significant yield potential, boasting an average harvest weight of 1007.5 kg. However, the remarkably high standard deviation of 1835.615 points to substantial disparities in soybean yields among individual farmers. This variability may stem from differences in cultivation practices, access to quality seeds, or the adoption of specific agricultural techniques tailored for soybean production. These findings not only underscore the crop preferences of the surveyed farmers but also highlight the need for targeted interventions and support measures to address the observed yield variations. By identifying and addressing the underlying factors contributing to these disparities, efforts can be made to enhance agricultural productivity, promote sustainable farming practices, and ultimately improve the livelihoods of smallholder farmers in the region.

**Table 5.** Number of climate smart agriculture practices adopted by smallholder farmers.

No._CSA_Adopted/Plot	Freq.	Percent	Cum.
0	167	49.26	49.26
1	123	36.28	85.55
2	43	12.68	98.23
3	4	1.18	99.41
4	1	0.29	99.71
5	1	0.29	100
**Total**	**339**	**100**	

**Table 6.** Quantities of crops harvested.

Variable	Obs	Mean	Std. Dev.	Min	Max
All Crops	339	1223.51	1442.82	50	9450
Maize	173	1766.57	1594.23	165	9450
Groundnuts	85	511.08	605.07	50	3450
Sunflower	61	609.6721	513.0212	50	2800
Soya beans	14	1007.5	1835.615	200	7245

**3.1.6 Productivity of various crops (Yield (kg) per hectare)**

The study’s findings provide valuable insights into crop productivity levels among the surveyed farming communities. For overall crop yield across all types, the average yield per hectare was 1,316.60 kg, though a substantial standard deviation of 1,214.13 suggests considerable variability in individual yields ( Table 7). For maize, a vital staple crop, the mean yield per hectare was calculated at 1,682.52 kg, with a high standard deviation of 1,325.87. This substantial disparity in maize yields may result from varying factors such as soil fertility, farming techniques, and environmental conditions. Groundnuts, another significant regional crop, had an average yield of 822.90 kg per hectare, with a standard deviation of 547.88. These variations likely reflect challenges in optimizing groundnut production, including pest and disease pressures, access to quality inputs, and possible knowledge gaps. Sunflower yields averaged 962.79 kg per hectare, showing a relatively lower standard deviation of 437.38. This consistency may be attributed to well-suited cultivation practices or more uniform environmental conditions that favour sunflower growth. Soybeans, a high-demand crop in the region, averaged 808.40 kg per hectare, with a moderate standard deviation of 426.74, suggesting that variations in yield could be influenced by factors such as seed quality, planting methods, and soil management practices. These findings not only offer an overview of productivity levels for various crops but also underscore the importance of targeted interventions to address yield disparities. By identifying and mitigating the underlying factors contributing to these differences, efforts can be made to improve agricultural productivity, support sustainable farming practices, and enhance the livelihoods of smallholder farmers in the region.

**Table 7.** Productivity of various crops (yield (kg) per hectare).

Yield per hectare (Kg)	Obs	Mean	Std. Dev.	Min	Max
All Crops	339	1316.60	1214.13	106.67	11630.67
Maize	173	1682.54	1325.87	119.00	11630.67
Groundnuts	85	822.9003	547.8818	106.6667	2500
Sunflower	61	962.7869	437.3807	300	2000
Soya beans	14	808.4048	426.7391	200	1740

**3.1.7 Impact of climate-smart practices on crop productivity among smallholder farmers**

The study examined the impact of Climate-Smart Agriculture (CSA) techniques on the crop yields of smallholder farmers. It was found that farmers who adopted CSA practices experienced a 20.20% higher crop yield compared to those who did not adopt these practices ( Table 8). The difference was statistically significant, with a *p*-value of 0.027 ( *p* < 0.05). These results suggest that the adoption of CSA practices leads to increased crop yields.

**Table 8.** Impact of climate-smart practices on crop productivity among smallholder farmers.

Treatment-effects estimation Number of Obs = 194					
Estimator: propensity-score matching Matches: requested = 1					
Outcome model: matching min = 1					
Treatment model: logit max = 2					
log_yield	Coef.	AI Robust Std. Err.	Z	P>z	[95% Conf. Interval]
ATE					
CSA_Practice					
(Adopters vs Non_Adopters)	.2019652	.0911943	2.21	0.027 **	.0232276–.3807028

Where, CSA = climate-smart agriculture, ATE = average treatment effect, Significance codes:

*** <1%,

** <5% and

* <10%; Author’s calculation using Stata 15MP.

**3.1.8 Impact of climate-smart practices on maize productivity among smallholder farmers**

The study utilized propensity score matching analysis to specifically assess the impact of CSA on maize productivity ( Table 9). The findings indicated that CSA adoption led to a 21.50% increase in maize yield compared to non-adoption. This significant increase in maize yield, with a *p*-value of 0.035 ( *p* < 0.05), demonstrates the positive effect of CSA practices on maize productivity.

**Table 9.** Impact of climate-smart practices on maize productivity among smallholder farmers.

Treatment-effects estimation Number of Obs = 194					
Estimator: propensity-score matching Matches: requested = 1					
Outcome model: matching min = 1					
Treatment model: logit max = 1					
log_yield	Coef.	AI Robust Std. Err.	Z	P>z	[95% Conf. Interval]
ATE					
CSA_Practice					
(Adopters vs Non_Adopters)	0.215012	0.101795	2.11	0.035 **	0.015496–0.414527

Where, CSA = climate-smart agriculture, ATE = average treatment, Significance codes:

*** <1%,

** <5% and

* <10%; Authors’calculation using Stata 15MP.

**3.1.9 Factors affecting smallholder farmers’ adoption of climate-smart agricultural**

The study examines the factors influencing the adoption of Climate-Smart Agriculture (CSA) practices among smallholder farmers using logistic regression analysis. The results reveal several key drivers of CSA adoption, as well as factors that do not significantly impact adoption rates. Age and farming experience emerge as the most significant positive factors affecting CSA adoption. Age has a strong positive and statistically significant effect (p = 0.000), indicating that older farmers are more likely to adopt CSA practices, likely due to their greater experience and awareness of climate risks ( Table 10). Similarly, farming experience shows a strong positive impact (p = 0.000), suggesting that farmers with more years of experience are more familiar with agricultural challenges and solutions, making them more inclined to adopt CSA practices.

**Table 10.** Factors affecting smallholder farmers’ adoption of climate-smart agricultural practices.

Logistic regressionNumber of Obs = 194						
Wald chi ^2^(10) = 27.34						
Prob > chi ^2^ = 0.0112						
Log Pseudolikelihood = -204.0124 Pseudo R ^2^ = 0.0965						
CSA_Practice	Coef.	Robust Std. Err.	z	P>z	[95% Conf. Interval]	
Age	0.0857 ***	0.0222	3.86	0.000	0.0422	0.1292
Gender	0.0173 *	0.4056	0.44	0.066	-0.7776	0.8122
Marital_status	-0.1788	0.1399	-1.28	0.201	-0.4530	0.0955
Education	-0.0510	0.0387	-1.32	0.187	-0.1270	0.0249
Farming_experience	0.0871 ***	0.0200	4.36	0.000	0.0480	0.1263
Household_size	-0.0279	0.0658	-0.42	0.672	-0.1569	0.1011
Income	0.000035 *	0.0000	1.85	0.064	0.0000	0.0001
Fertilizer	0.0007	0.0007	1.12	0.263	-0.0005	0.0020
Farm_size	-0.0201 **	0.0449	-0.45	0.005	-0.1082	0.0680
Livestockqt	0.0067 *	0.0083	0.81	0.018	-0.0230	0.0095
Credit_access	-0.1508	0.2405	-0.63	0.531	-0.6221	0.3205
Access_to_climate_inform	-0.4411 **	0.5920	-0.75	0.006	-1.6014	0.7192
Extension_services	-0.0181	0.2964	-0.06	0.951	-0.5989	0.5628
_cons	-0.4161	1.0016	-0.42	0.678	-2.3792	1.5470

Significance codes:

*** <1%,

** <5% and

* <10%; Author’s calculation using Stata 15MP.

On the other hand, farm size and access to climate information have significant negative effects on CSA adoption. Farm size has a small but statistically significant negative effect (p = 0.005), implying that smaller farms are more likely to adopt CSA practices, possibly due to greater flexibility or resource constraints. Access to climate information also has a strong negative effect (p = 0.006), which is counterintuitive. This may indicate that farmers with access to climate information face barriers, such as a lack of resources, trust, or understanding, that prevent them from adopting CSA practices.

Several factors have only marginally significant or insignificant effects on CSA adoption. Gender, income, and livestock quantity show small positive effects, but the evidence is weak. For instance, gender has a marginally significant positive effect (p = 0.066), suggesting that male farmers may be slightly more likely to adopt CSA practices than female farmers. Income also has a marginally significant positive effect (p = 0.064), indicating that higher income may enable farmers to invest in CSA practices, though the effect is not strong. Livestock ownership has a small positive effect (p = 0.018), possibly because livestock provide additional resources or incentives for adopting CSA practices. However, factors such as marital status, education, household size, fertilizer use, credit access, and extension services do not significantly influence CSA adoption.

The findings of this study have important policy implications. First, efforts should focus on leveraging the knowledge and experience of older and more experienced farmers to promote CSA adoption. Second, smaller farms, which are more likely to adopt CSA practices, should be targeted with tailored support to enhance adoption rates. Third, the negative effect of access to climate information highlights the need to address barriers that prevent farmers from translating information into action, such as lack of resources, trust, or understanding. Fourth, while extension services did not show a significant impact, their effectiveness could be improved through better training and resource allocation to promote CSA practices. Finally, promoting livestock ownership may provide additional incentives for adopting CSA practices, given its marginally significant positive effect.

**3.1.10 Factors affecting smallholder farmers’ crop productivity**

A Cobb-Douglas production function analysis was employed to examine the determinants of agricultural yield ( Table 11). The study examines the factors influencing crop productivity among smallholder farmers using linear regression analysis. The results reveal several key drivers of crop productivity, as well as factors that do not significantly impact yields. Fertilizer use, livestock quantity, and income emerge as the most significant positive factors affecting crop productivity. Fertilizer use, in particular, has a strong and statistically significant positive effect (p = 0.000), indicating that increased application of fertilizers is associated with higher crop yields. Similarly, livestock ownership shows a strong positive impact (p = 0.000), likely due to the benefits of manure for soil fertility and additional income for farm investments. Income also has a small but statistically significant positive effect (p = 0.004), suggesting that higher income enables farmers to invest in better inputs and technologies, thereby improving productivity.

**Table 11.** Factors affecting smallholder farmers’ crop productivity.

Linear regression Number of Obs = 194						
F(9, 179) = 11.05						
Prob > F = 0.0000						
R-squared = 0.6441						
Root MSE = 0.74495						
log_yield	Coef.	Robust Std. Err.	t	P>t	[95% Conf. Interval]	
Age	-0.0019	0.0051	-0.37	0.709	-0.0120	0.0082
Gender	0.0385	0.1126	0.34	0.732	-0.1830	0.2601
Marital_status	0.0076 *	0.0379	0.20	0.841	-0.0670	0.0821
Education	0.0098 *	0.0122	0.80	0.422	-0.0142	0.0338
Farming_experience	0.0043	0.0049	0.88	0.377	-0.0053	0.0140
Household_size	0.0231 **	0.0181	1.28	0.201	-0.0124	0.0586
Income	0.00002 **	0.0000	2.94	0.004	0.0000	0.0000
Fertilizer	0.0012 ***	0.0002	8.13	0.000	0.0009	0.0015
Farm_size	-0.0652 ***	0.0145	-4.49	0.000	-0.0938	-0.0366
Livestockqt	0.0086 ***	0.0018	4.79	0.000	0.0051	0.0122
CSA_Practice	0.1349 *	0.0747	1.81	0.072	-0.0120	0.2818
Credit_access	-0.1171	0.0741	-1.58	0.115	-0.2629	0.0287
Access_to_climate_info.	-0.1523	0.1974	-0.77	0.441	-0.5408	0.2361
Extension_services	0.0429	0.0846	0.51	0.612	-0.1236	0.2094
_cons	7.1936 ***	0.3095	23.24	0.000	6.5845	7.8026

Significance codes:

*** <1%,

** <5% and

* <10%; Author’s calculation using Stata 15MP.

On the other hand, farm size has a significant negative effect on crop productivity (p = 0.000), implying that smaller farms tend to be more productive per unit area compared to larger farms. This could be due to more efficient resource allocation or better management practices on smaller farms. Household size also has a positive and statistically significant effect (p = 0.201), possibly because larger households provide more labour for farming activities, leading to higher yields.

However, several factors were found to have no significant impact on crop productivity. These include age, gender, marital status, education, farming experience, credit access, access to climate information, and extension services. For instance, access to credit and extension services, often considered critical for agricultural development, did not show a significant positive effect in this study. This could indicate that these resources are not being effectively utilized or are not adequately tailored to the needs of smallholder farmers in this context. Similarly, access to climate information did not significantly improve yields, suggesting that information dissemination alone may not be sufficient to enhance productivity without complementary support.

The findings of this study have important policy implications. First, efforts should focus on improving access to and affordability of fertilizers, as their use is strongly associated with higher crop yields. Second, promoting livestock ownership can enhance productivity through the dual benefits of manure for soil fertility and additional income for farm investments. Third, smaller farms, which tend to be more productive per unit area, should be targeted with tailored support to maintain their efficiency. Finally, while extension services and credit access did not show significant impacts in this study, their effectiveness could be improved through better training, resource allocation, and alignment with the specific needs of smallholder farmers. By addressing these factors, policymakers and development practitioners can help smallholder farmers achieve higher crop productivity and improve their livelihoods.

**3.1.11 Discussion of the results**

The study investigated the effects of Climate-Smart Agricultural (CSA) practices on crop productivity among smallholder farmers in the Nyimba district of Zambia and explored the factors that influence both the adoption of CSA practices and crop production in the area. The analysis showed that smallholder farmers implementing CSA methods achieved a 20.20% higher overall crop yield than those not using these practices. Specifically, CSA adopters saw a 21.50% increase in maize yield compared to non-adopters. These findings highlight the potential of CSA practices to substantially improve crop productivity among smallholder farmers. The observed yield increases suggest that climate-smart methods could serve as an effective strategy for enhancing agricultural productivity and strengthening food security in the region.

This study aligns with findings by Zakaria et al. (2020), who identified that factors such as rice cultivation experience, media and training access, and perceived reductions in rainfall positively influenced the adoption of climate-smart agricultural (CSA) technologies. Conversely, factors such as larger farm sizes, greater distances between farmers’ residences and fields, location, and increased temperatures were found to deter CSA adoption. The present study, however, emphasizes age and income as facilitators of CSA adoption, diverging from Saha et al. (2019), who highlighted education, occupation, family size, farm size, climate adaptation methods, cattle ownership, market access, information access, training, organizational affiliations, and climate change perceptions as significant determinants. This contrast suggests that while higher income facilitates CSA adoption, other factors—such as farm size, accessibility, and climatic perceptions—may act as barriers.

Supporting these findings, Kurgat et al. (2020) reported that female ownership of assets, farm location, and household resources significantly affected CSA adoption in Tanzania. Similarly, Aryal et al. (2018) identified household characteristics, market access, and primary climate hazards as factors influencing the probability and extent of CSA adoption. On the other hand, Abegunde et al. (2019) found no significant impact from marital status, education, fertilizer use, credit access, or extension services on CSA adoption, highlighting the complexity of CSA uptake across regions. The study by Urgessa (2015) underscored additional factors influencing agricultural labour and crop productivity, such as the land-labour ratio, pesticide and fertilizer use, manure application, and household size. WenJing et al. (2021) observed income disparities among households, finding that middle- and upper-middle-class households benefited more from renting out farmland, while households with high on-farm income were less inclined to expand through land rental. Similarly, Du et al. (2020) demonstrated that synthetic fertilizer and manure applications positively affect soil productivity and crop yields. Furthermore, livestock contributes both manure and income, which can be reinvested in crop-enhancing technologies ( Anderson, 1989; Beedy et al., 2010).

Remarkably, this study affirms that CSA adoption favourably impacts crop productivity. Supporting these findings, Mujeyi et al. (2021) reported that CSA practices significantly increased crop yields for smallholder farmers in integrated crop-livestock systems. This conclusion aligns with Serote et al. (2021), who found that household demographics and institutional factors influence CSA adoption and subsequent productivity outcomes. Together, these studies emphasize the interplay of factors shaping agricultural outcomes and the importance of targeted interventions to enhance productivity and sustainability. Addressing drivers such as farm size, livestock integration, and demographic characteristics could enable policymakers to support smallholder farmers in achieving better yields and environmental resilience.

Multiple studies also highlight the broader impacts of CSA practices on productivity, food security, and soil health. Abegunde et al. (2022) found that CSA adoption favourably affected food security among 327 Nigerian smallholders, with agricultural and non-farm income both contributing to this outcome. Similarly, Mossie (2022) found that CSA technology, specifically wheat row planting, significantly improved productivity in Southern Ethiopia, with adopters achieving 1,368 kg more wheat per hectare than non-adopters. Tadesse et al. (2021) reported that CSA practices in Ethiopia positively impacted soil carbon, productivity, and fertility, while Kichamu-Wachira et al. (2021) concluded that CSA advances yield, soil carbon, and nitrogen levels, providing climate benefits and enhancing food production.

In addition, Amadu et al. (2020) found that 53% of CSA adopters in southern Malawi achieved higher maize yields during the 2016 drought, underscoring CSA’s resilience benefits. These studies collectively underscore the potential of CSA practices to improve crop productivity, food security, and soil health, fostering resilience among smallholder farmers amid climate variability. The present study reinforces the potential of CSA to increase yields and nutrient availability, aiding resource-poor farmers in adapting to climate change. These findings echo those of Kichamu-Wachira et al. (2021), who documented CSA’s positive effects on yields, soil carbon, and nitrogen levels in Africa. Further, Amadu et al. (2020) noted that CSA adoption during drought years contributed to increased maize yields among Malawian smallholders. Similarly, Fentie and Beyene (2019) found a positive effect of CSA adoption on crop yield per hectare, underscoring CSA’s potential to improve productivity, food security, and soil quality for smallholder farmers in the face of climate change.

## 4. Conclusion

This study examined the factors influencing crop productivity and the adoption of Climate-Smart Agriculture (CSA) practices among smallholder farmers. The findings reveal that fertilizer use, livestock ownership, and income are the most significant drivers of increased crop productivity, highlighting the importance of access to agricultural inputs and financial resources. Conversely, larger farm sizes were associated with lower productivity, suggesting that smaller farms may be more efficient per unit area. Factors such as age, gender, education, and access to extension services did not significantly influence productivity, indicating a need for more targeted and effective support systems. In terms of CSA adoption, older and more experienced farmers were more likely to adopt CSA practices, likely due to their greater awareness of climate risks and agricultural challenges. However, access to climate information had a counterintuitive negative effect, suggesting barriers such as lack of resources or trust may hinder adoption. Smaller farms were also more likely to adopt CSA practices, possibly due to greater flexibility. Factors like credit access and extension services did not significantly influence CSA adoption, pointing to gaps in their effectiveness. The study underscores the need for targeted interventions to enhance productivity and CSA adoption. Policies should focus on improving access to fertilizers, promoting livestock ownership, and supporting smaller farms. Addressing barriers to the use of climate information and strengthening extension services are also critical. By leveraging these insights, policymakers can design strategies to improve agricultural resilience, boost productivity, and support sustainable farming practices among smallholder farmers, ultimately enhancing their livelihoods and food security.

## Ethics and consent

The study was approved by the University of Zambia Directorate of Research and Graduate Studies (NASREC) on March 18, 2024, and is registered under NASREC IRB No. 00005465 (IORG No. 0005376). The principal researcher provided a written consent form for research participants to participate in the household survey, conforming to the research ethics guidelines of the University of Zambia and Haramaya University.

## Author contributions

Authors contributed in the following ways: “Conceptualization, C.P. and F.S.; methodology, C.P.; software, C.P.; validation, S.C., F.S.; formal analysis, C.P.; investigation, C.P.; resources, S.M.; data curation, C.P.; writing—original draft preparation, C.P.; writing—review and editing, S.M.; supervision, F.S. and S.C. All authors have read and agreed to the published version of the manuscript.” Authorship must be limited to those who have contributed substantially to the work reported.

## Data Availability

Zenodo: Adoption of Climate-Smart Agricultural Practices and Its Impact on Crop Productivity: A Case Study of Smallholder Farmers in Nyimba District, Zambia [Data set]. Zenodo. https://doi.org/10.5281/zenodo.11258819 ( Chavula, P., et al., 2024) This project contains the following underlying data:
•
Edited_Survey data 28.03.2022.dta Data are available under the terms of the Creative Commons Attribution 4.0 International

## References

[ref1] Abegunde VO Sibanda M Obi A : The dynamics of climate change adaptation in sub-Saharan Africa: A review of climate-smart agriculture among small-scale farmers. *Climate.* 2019;7(11). 10.3390/cli7110132

[ref2] Abegunde VO Sibanda M Obi A : Effect of climate-smart agriculture on household food security in small-scale production systems: A micro-level analysis from South Africa. *Cogent Social Sciences.* 2022;8(1):2086343. 10.1080/23311886.2022.2086343

[ref3] Alfani F Arslan A McCarthy N : Climate-change vulnerability in rural Zambia: the impact of an El Niño-induced shock on income and productivity. *ESA Working Paper.* 2019.

[ref45] Ali A Abdulai A : The adoption of genetically modified cotton and poverty reduction in Pakistan. *J. Agric. Econ.* 2010;61:175–192. 10.1111/j.1477-9552.2009.00227.x

[ref4] Amadu FO Miller DC McNamara PE : Agroforestry as a pathway to agricultural yield impacts in climate-smart agriculture investments: Evidence from southern Malawi. *Ecol. Econ.* 2020;167(October 2018):106443. 10.1016/j.ecolecon.2019.106443

[ref5] Anderson JM : Tropical Soil Biology and Fertility Methods_Web Soils Reading.pdf. 1989;171. J. S. I. I.

[ref48] Arslan A McCarthy N Lipper L : Adoption and intensity of adoption of conservation farming practices in Zambia. *Agric. Ecosyst. Environ.* 2014;187:72–86. 10.1016/j.agee.2013.08.017

[ref6] Aryal JP Rahut DB Maharjan S : Factors affecting the adoption of multiple climate-smart agricultural practices in the Indo-Gangetic Plains of India. *Nat. Res. Forum.* 2018;42(3):141–158. 10.1111/1477-8947.12152

[ref41] Baker M Melino A : Duration dependence and nonparametric heterogeneity: A Monte Carlo study. *J. Econom.* 2000;96:357–393. 10.1016/S0304-4076(99)00064-0

[ref7] Beedy TL Snapp SS Akinnifesi FK : Impact of Gliricidia sepium intercropping on soil organic matter fractions in a maize-based cropping system. *Agric. Ecosyst. Environ.* 2010;138(3–4):139–146. 10.1016/j.agee.2010.04.008

[ref46] Branca G Paolantonio A Cavatassi R : Climate-Smart Agriculture Practices in Zambia. 2016.

[ref40] Caliendo M Kopeinig S : Some practical guidance for the implementation of propensity score matching. *J. Econ. Surv.* 2008;22(1):31–72. 10.1111/j.1467-6419.2007.00527.x

[ref8] Chavula P : A Review between Climate Smart Agriculture Technology Objectives’ Synergies and Tradeoffs. *Int. J. Agric. Food Sci.* 2021;5(4):748–753. 10.26855/ijfsa.2021.12.023

[ref9] Chavula P : An Overview of Challenges that Negatively Affect Agriculture Performance in Sub-Sahara Africa: Synthesis Study. 2022;6(11):261–269.

[ref10] Chavula P Feyissa S Sileshi M : Adoption of Climate-Smart Agricultural Practices and Its Impact on Crop Productivity: A Case Study of Smallholder Farmers in Nyimba District, Zambia.[Data set]. *Zenodo.* 2024. 10.5281/zenodo.11258819 PMC12371323 40860262

[ref11] CIAT, & World Bank: Climate-Smart Agriculture in Zambia. *CSA Country Profiles for Africa Series.* 2017.

[ref12] Du Y Cui B Zhang Q : Effects of manure fertilizer on crop yield and soil properties in China: A meta-analysis. *Catena.* 2020;193:104617. 10.1016/j.catena.2020.104617

[ref13] Fentie A Beyene AD : Climate-smart agricultural practices and welfare of rural smallholders in Ethiopia: Does planting method matter? *Land Use Policy.* 2019;85:387–396. 10.1016/j.landusepol.2019.04.020

[ref44] Gujarati DN : *Basic Econometrics.* International Third ed. New York: McGraw-Hill, Inc.;1995.

[ref14] Gumbo DJ Dumas-Johansen M : The role of miombo woodlands in the three Rio conventions. *Clim. Dev.* 2021;13(2):107–114. 10.1080/17565529.2020.1729686

[ref15] Halperin J Lemay V Chidumayo E : Model-based estimation of above-ground biomass in the miombo ecoregion of Zambia. *Forest Ecosystems.* 2016;3:1–17. 10.1186/s40663-016-0077-4

[ref16] IPCC: Global warming of 1.5°C. An IPCC Special Report on the impacts of global warming of 1.5°C above pre-industrial levels and related global greenhouse gas emission pathways, in the context of strengthening the global response to the threat of climate change. *Ipcc - Sr15.* 2018;2(October):17–20. Reference Source

[ref17] Ivanova D Barrett J Wiedenhofer D : Quantifying the potential for climate change mitigation of consumption options. *Environ. Res. Lett.* 2020;15(9):93001. 10.1088/1748-9326/ab8589

[ref47] Kaczan D Arslan A Lipper L : Climate-smart agriculture? A review of current practice of agroforestry and conservation agriculture in Malawi and Zambia. 2013.

[ref18] Kalinda T : Use of integrated land use assessment (ilua) use of integrated land use data for forestry and agriculture. 2008.

[ref19] Kichamu-Wachira E Xu Z Reardon-Smith K : Effects of climate-smart agricultural practices on crop yields, soil carbon, and nitrogen pools in Africa: a meta-analysis. *J. Soils Sediments.* 2021;21(4):1587–1597. 10.1007/s11368-021-02885-3

[ref20] Kurgat BK Lamanna C Kimaro A : Adoption of Climate-Smart Agriculture Technologies in Tanzania. *Front. Sustain. Food Syst.* 2020;4(May). 10.3389/fsufs.2020.00055

[ref21] Makate C : Local institutions and indigenous knowledge in adoption and scaling of climate-smart agricultural innovations among sub-Saharan smallholder farmers. 2019;270–287. 10.1108/IJCCSM-07-2018-0055

[ref22] Molieleng L Fourie P Nwafor I : Adoption of Climate Smart Agriculture by Communal Livestock Farmers in South Africa. *Sustainability.* 2021;13(18):10468. 10.3390/su131810468

[ref23] Montfort F Nourtier M Grinand C : Regeneration capacities of woody species biodiversity and soil properties in Miombo woodland after slash-and-burn agriculture in Mozambique. *For. Ecol. Manag.* 2021;488:119039. 10.1016/j.foreco.2021.119039

[ref24] Mossie WA : The Impact of Climate-Smart Agriculture Technology on Productivity: Does Row Planting Matter? Evidence from Southern Ethiopia. *Sci. World J.* 2022;2022:1–11. 10.1155/2022/3218287 35991088 PMC9391145

[ref25] Mujeyi A Mudhara M Mutenje M : The impact of climate smart agriculture on household welfare in smallholder integrated crop–livestock farming systems: evidence from Zimbabwe. *Agric. Food Secur.* 2021;10(1):1–15. 10.1186/s40066-020-00277-3

[ref26] Murray V Ebi KL : IPCC special report on managing the risks of extreme events and disasters to advance climate change adaptation (SREX). *J. Epidemiol. Community Health.* 2012;66(9):759–760. BMJ Publishing Group Ltd. 10.1136/jech-2012-201045, PMID:22766781

[ref27] Newell P Taylor O Naess LO : Climate smart agriculture? Governing the sustainable development goals in Sub-Saharan Africa. *Front. Sustain. Food Syst.* 2019;3:55. 10.3389/fsufs.2019.00055

[ref28] Ngoma H Lupiya P Kabisa M : Impacts of climate change on agriculture and household welfare in Zambia: an economy-wide analysis. *Clim. Chang.* 2021;167(3):1–20. 10.1007/s10584-021-03168-z

[ref29] Odubote IK Ajayi OC : Scaling Up climate-smart agricultural (CSA) solutions for smallholder Cereals and livestock farmers in Zambia. *Handbook of Climate Change Resilience.* 2020; pp.1115–1136. 10.1007/978-3-319-93336-8_109

[ref43] Pindyck RS Rubinfeld DL : *Econometric Models and Economic Forecasts.* New York: McGraw-Hill;1981.

[ref30] Policy brief:2016. December, 1–6.

[ref42] Ravallion M : Evaluating antipoverty programs. *Handbook of development economics.* Vol.4.2007; pp.3787–3846. 10.1016/S1573-4471(07)04059-4

[ref31] Saha MK Abdul A Biswas A : Factors Affecting to Adoption of Climate-smart Agriculture Practices by Coastal Farmers’ in Bangladesh. *Am. J. Environ. Sustain. Dev.* 2019;4(4):113–121.

[ref32] Serote B Mokgehle S Plooy C Du : Factors influencing the adoption of climate-smart irrigation technologies for sustainable crop productivity by smallholder farmers in arid areas of South Africa. *Agriculture (Switzerland).* 2021;11(12). 10.3390/agriculture11121222

[ref33] Sharifi A : Co-benefits and synergies between urban climate change mitigation and adaptation measures: A literature review. *Sci. Total Environ.* 2021;750:141642. 10.1016/j.scitotenv.2020.141642 32858298

[ref38] Stahlbaumer L Janicke R Hache I : Climate change effects on agronomic practices and livelihood. *Agric. Sci.* 2022;3:20.

[ref34] Tadesse M Simane B Abera W : The effect of climate-smart agriculture on soil fertility, crop yield, and soil carbon in southern ethiopia. *Sustainability.* 2021;13(8):4515. 10.3390/su13084515

[ref39] Timberlake J Chidumayo E Sawadogo L : Distribution and characteristics of African dry forests and woodlands. The dry forests and woodlands of Africa. Routledge;2010; pp.11–41.

[ref35] Urgessa T : The Determinants of Agricultural Productivity and Rural Household Income in Ethiopia. *Ethiop. J. Econ.* 2015;24(2):63–91.

[ref36] WenJing H ZhengFeng Z XiaoLing Z : Farmland Rental Participation, Agricultural Productivity, and Household Income: Evidence From Rural China. *Land.* 2021;10(9):1–22.

[ref37] Zakaria A Alhassan SI Kuwornu JKM : Factors Influencing the Adoption of Climate-Smart Agricultural Technologies Among Rice Farmers in Northern Ghana. *Earth Syst. Environ.* 2020;4(1):257–271. 10.1007/s41748-020-00146-w

