# Supplementary material for: Factors Influencing Climate-Smart Agriculture Practices Adoption and Crop Productivity among Smallholder Farmers in Nyimba District, Zambia
Source: F1000Res. 2025 Mar 21;13:815. Originally published 2024 Jul 19. [Version 3] doi: 10.12688/f1000research.144332.3 (PMC12371323; doi:10.12688/f1000research.144332.3)
Supplement: Peer review history

# Reviewer report: Reviewer response for version 3

**DOI:** 10.5256/f1000research.178749.r396180

**Published:** 2025-08-23

**Authors:** Krishan Verma

**Affiliations:** 1 Guangxi Academy of Agricultural Sciences, Nanning, China

In the present article authors discussed the role of climate smart agriculture strategies adoption and crop productivity among smallholder farmers in the district of Nyimba, Zambia. It is very important issue in the era of climate change. 

 The authors incorporated all comments/ suggestions very nicely. No further corrections are required. The article can be accepted in its present form.

Is the work clearly and accurately presented and does it cite the current literature?

Yes

If applicable, is the statistical analysis and its interpretation appropriate?

Yes

Are all the source data underlying the results available to ensure full reproducibility?

Yes

Is the study design appropriate and is the work technically sound?

Yes

Are the conclusions drawn adequately supported by the results?

Yes

Are sufficient details of methods and analysis provided to allow replication by others?

Yes

Reviewer Expertise:

Abiotic and biotic stresses, Environmental eco-physiology

I confirm that I have read this submission and believe that I have an appropriate level of expertise to confirm that it is of an acceptable scientific standard.

# Reviewer report: Reviewer response for version 3

**DOI:** 10.5256/f1000research.178749.r397795

**Published:** 2025-08-21

**Authors:** Imaneh Goli

**Affiliations:** 1 University of Liège, Gembloux, Belgium

Title of the manuscript: Factors Influencing Climate-Smart Agriculture Practices Adoption and Crop Productivity among Smallholder Farmers in Nyimba District, Zambia

 This study examines the factors influencing smart farming practices. The authors have attempted to address important aspects such as the impact of climate change on agriculture and the necessity of climate-smart agriculture (CSA). However, the study has several scientific and structural weaknesses. Addressing the following comments would help improve this study:

 Abstract

1. Page 1: *"Climate change significantly affects smallholder farmers, whose livelihoods are closely tied to the environment."* Please continue this sentence by stating the study gap and briefly explaining which challenges led you to investigate the factors and drivers of smart agriculture adoption in this study.
2. Page 1: *"Data were collected from 194 households across 12 villages, and logistic regression and propensity score matching analyses were employed to identify key factors and evaluate CSA’s effects on crop yields."* Please explain what the sampling method was?
3. Page 1: *"Findings revealed that CSA adoption is influenced by factors such as education level, household size, fertilizer use, age, gender, farming experience, livestock ownership, income, farmland size, marital status, and access to climate-related information."* Please explain which of these factors has the greatest impact on the adoption of smart agriculture among farmers.
4. Also, it is not clear from the above sentence whether these factors have a positive or negative effect? The authors should explain the results more clearly.
5. Page 1: *"The study underscores the need for targeted interventions to support CSA adoption through education, improved dissemination of climate information, and access to critical resources such as improved seeds and financial services."* Providing practical solutions for CSA (e.g. mulching, intercropping) could help improve the applicability of this study. Therefore, please revise this sentence with practical solutions in mind.

Introduction:

1. Most of the explanations provided in the introduction section address the issue of climate change and shocks and do not explain why and how CSA can adapt to climate change and increase productivity. Therefore, authors should strengthen the introduction section by providing explanations of CSA and making connections between CSA and climate change.
2. It would be better for authors to mention the types of CSA methods in the introduction and explain which methods have the greatest impact in addressing climate change.
3. Page 3: *"Climate change is projected to contribute to and/or is already causing a global reduction in cereal yields, such as maize and wheat declining by 3.8% and 5.5% respectively (Branca etal., 2016; Ngoma et al.,2021)."* Can CSA really offset these losses? In this context, it would be better for the authors to point to studies that have measured the reduction in climate losses with CSA.
4. In the introduction, the authors did not explain what the significance of this study is and why this particular study was necessary. Please clarify this at the end of the introduction. First, identify the gaps in the study and explain what previous research was lacking that this study addresses.
5. Also, this study lacks innovation. The main innovation of this study is not clear. Please clearly identify the innovation of the study and state what added value this study has globally.
6. The aim/aims of the study are not clearly stated. Please specify which aim/aims this study was conducted against.

Conceptual framework:

1. Page 5: *"1.1 Conceptual frameworks: In the face of changing climatic conditions and climate change, climate-smart agriculture (CSA) is an approach to transform and reorient agricultural systems to ensure food security (Chavula, 2021). Climate change disrupts food markets, putting food supply and production at risk. Strengthening farmers’ adaptive capacity and enhancing the mitigation potential and efficiency of agricultural production systems can mitigate these risks. Smallholder farmers who are aware of climate change or perceive it as a reality are more likely to adopt climate-smart agricultural practices. These farmers aim to achieve the three pillars of CSA: improving household income and productivity, enhancing resilience and adaptation, and reducing greenhouse gas emissions. The adoption of climate-smart agriculture to attain these principles is influenced by institutional, cognitive, and socioeconomic factors (Figure 1)."* The authors have only mentioned one reference in the theoretical framework section, which is not enough. Please strengthen this section and provide more information about the research framework. Also, this section mentions three pillars of CSA, but there is not enough explanation about it. Please explain more about the pillars of CSA and show these pillars in Figure 1.
2. Please move Figure 1 to the end of this sub-section ( *1.1 Conceptual frameworks*) and include the source of the figure. Also, provide a further explanation of Figure 1 and explain how the different components of Figure 1 relate to each other.

Methodology:

1. Page 7: *"To determine an appropriate sample size, the study employed Slovin’s formula. Additionally, three villages from the four selected camps were randomly chosen for data collection. These villages were Sikwenda, Sichipale, Mawanda, Elina, Katumbila, Sichalika, Malalo, Mwenecisango, Mulivi, Lengwe, Mofu, and Yona. With a margin of error set at 0.05, the initial calculated sample size was 386 participants. However, to optimize resources, the researchers increased the margin of error to 0.1, resulting in a smaller sample size of 99 participants. Achieving this reduced sample size required additional time and financial investments, but it was a trade-off deemed necessary for the successful completion of the study."* This sampling section needs further explanation and clarification. Why did the authors initially calculate a sample size of 386, then reduce it to 99, and finally select 194? These changes were made without providing a convincing statistical justification, which is not correct. Also, the authors should explain with logical reasons why the margin of error was changed (from 0.05 to 0.1).
2. Please explain the validity and reliability of the questionnaire and explain the results of pre-tests and the changes made to them.
3. Please explain whether a standard questionnaire was used in this study or a researcher-made questionnaire? And explain the main focus of the questionnaire questions.
4. Please clarify why important variables such as soil quality, water availability, and annual climate changes that affect agricultural adaptation were not controlled for in the analysis.
5. Please elaborate on the statistical tests used and describe the steps involved in implementing the equilibrium test.

Results and Discussion:

1. Please present the results and discussion sections separately. In the results section, present the results of the main research analyses/tables/figures and their explanations. And in the discussion section, provide an in-depth interpretation of the findings and state what differences and similarities there are between the findings of this study and the findings of other studies.
2. The results in Table 7 show that the maximum yield of corn is much higher, but no explanation is given for this. Please provide the reasons for the increased corn yield in the explanation of Table 7.
3. Please report the matching results in full and provide more details on the balance diagnostics.

Conclusion:

1. The conclusion section needs substantial strengthening. Please refrain from repeating the results you have already explained in the results section in the conclusion section. Please provide a final and general conclusion in this section and explain the theoretical and methodological implications of this study.
2. Provide several practical and implementable solutions to improve the current situation in the conclusion section. Try to make the solutions at the level of local farmers who can easily adapt to climate change.
3. Also, point out the limitations of this study and explain how future studies can address these limitations and introduce several perspectives and topics for future studies.

Is the work clearly and accurately presented and does it cite the current literature?

Yes

If applicable, is the statistical analysis and its interpretation appropriate?

Yes

Are all the source data underlying the results available to ensure full reproducibility?

Yes

Is the study design appropriate and is the work technically sound?

Yes

Are the conclusions drawn adequately supported by the results?

Partly

Are sufficient details of methods and analysis provided to allow replication by others?

Partly

Reviewer Expertise:

Climate-smart agriculture; Sustainable livelihood; Resilience

I confirm that I have read this submission and believe that I have an appropriate level of expertise to confirm that it is of an acceptable scientific standard, however I have significant reservations, as outlined above.

# Reviewer report: Reviewer response for version 2

**DOI:** 10.5256/f1000research.174061.r364188

**Published:** 2025-02-20

**Authors:** Tinawaen Tambol

**Affiliations:** 1 West African Centre for Sustainable Rural Transformation (WAC-SRT), Abdou Moumouni University of Niamey, Niamey, Niger

Thank you for inviting me to review this paper on an important topic 'Factors Influencing Climate-Smart Agriculture Practices Adoption and Crop Productivity among Smallholder Farmers in Nyimba District, Zambia'.

 I hope the following comments and feedback will help improve the quality of the paper. 

**Introduction**

 In the second paragraph, 'Climate change is projected to contribute to and/or is already causing a global reduction in cereal yields, such as maize and wheat declining by 3.8% and 5.5%, respectively,' it is good to cite the source of the figures.

 Significant theoretical arguments could improve upon the background.

**Methods**

 The location is well described, but without referencing the source of the information.

 It will be best to indicate the source of weather information for replication. The vegetation is presented well. 

 The in-text citation lacks consistency. ( Baker and Melino, 2000),While others use 'ampersand'.

**Results**

 Overall, the study's results are well presented. 

**Discussion**

 The results were discussed concerning existing literature. 

 However, the limitation of the study is not articulated and the authors are silent on a direction for future studies.

**Conclusion**

 The conclusion is well-articulated with suggested recommendations. 

 GENERAL COMMENTS

 The manuscript is interesting, however, some shortcomings need to be addressed to improve it.

Is the work clearly and accurately presented and does it cite the current literature?

Yes

If applicable, is the statistical analysis and its interpretation appropriate?

Yes

Are all the source data underlying the results available to ensure full reproducibility?

Yes

Is the study design appropriate and is the work technically sound?

Yes

Are the conclusions drawn adequately supported by the results?

Yes

Are sufficient details of methods and analysis provided to allow replication by others?

Partly

Reviewer Expertise:

Climate Smart Agriculture Technologies, Rural and City planning, GIS, Climate Change and Environmental Management.

I confirm that I have read this submission and believe that I have an appropriate level of expertise to confirm that it is of an acceptable scientific standard, however I have significant reservations, as outlined above.

# Reviewer report: Reviewer response for version 2

**DOI:** 10.5256/f1000research.174061.r345496

**Published:** 2024-12-10

**Authors:** Rao Sabir Sattar

**Affiliations:** 1 University of Agriculture, Faisalabad, Pakistan

I have gone through the article, and changes have been made by the authors, which are satisfactory.

Is the work clearly and accurately presented and does it cite the current literature?

Partly

If applicable, is the statistical analysis and its interpretation appropriate?

Partly

Are all the source data underlying the results available to ensure full reproducibility?

Yes

Is the study design appropriate and is the work technically sound?

Yes

Are the conclusions drawn adequately supported by the results?

Yes

Are sufficient details of methods and analysis provided to allow replication by others?

Yes

Reviewer Expertise:

livelihood vulnerability, climate change adaptation, extension methods

I confirm that I have read this submission and believe that I have an appropriate level of expertise to confirm that it is of an acceptable scientific standard.

# Reviewer report: Reviewer response for version 1

**DOI:** 10.5256/f1000research.158112.r323712

**Published:** 2024-10-10

**Authors:** Rao Sabir Sattar

**Affiliations:** 1 University of Agriculture, Faisalabad, Pakistan

The article titled "Factors Influencing Climate-Smart Agriculture Practices Adoption and Crop Productivity among Smallholder Farmers in Nyimba District, Zambia" offers an insightful analysis into the drivers behind the adoption of climate-smart agricultural (CSA) practices and their effect on crop productivity. While the study is well-structured and addresses critical concerns of smallholder farmers in the context of climate change, there are, in my opinion, several areas of strength and opportunities for improvement.

 Strengths:

 The study's focus on climate-smart agriculture (CSA) adoption is highly pertinent, particularly given the increasing threats of climate change on smallholder farmers, whose livelihoods depend heavily on climate-sensitive agricultural activities. It fills a critical research gap by examining both the factors driving CSA adoption and the tangible benefits of CSA in terms of crop productivity.

 The use of logistic regression and propensity score matching is appropriate and well-explained. These methods provide robust insights into the causal relationship between CSA adoption and increased crop productivity, allowing the study to demonstrate clear statistical correlations between the independent and dependent variables.

 The study examines a wide array of factors influencing CSA adoption, such as education, household size, farming experience, and access to climate-related information. This thoroughness highlights the complexity of decision-making processes among smallholder farmers and presents a holistic view of the influencing factors.

 The study’s findings provide actionable insights for policymakers and stakeholders. The clear demonstration of the positive impact of CSA practices on crop productivity, especially maize yields, can inform policy interventions aimed at promoting CSA in regions similar to Nyimba District. Additionally, the focus on educational level and access to information emphasizes the need for targeted extension services.

 Areas for Improvement:

 While the article presents a detailed account of the findings, the discussion could benefit from deeper engagement with the implications of the results. For instance, how do the identified factors interact with one another, and are there synergies or trade-offs in adopting CSA practices? Further, it would be helpful to discuss the potential barriers that may prevent broader adoption of CSA practices, beyond those identified by the logistic regression model.

 The paper could strengthen its impact by better contextualizing the results within the broader literature on CSA adoption in Africa and other developing regions. A comparative analysis with other regions facing similar climate challenges would offer a more nuanced understanding of how the findings fit into the global or regional discourse on CSA.

 While the study hints at policy implications, the recommendations could be made more explicit. For example, based on the factors influencing CSA adoption, what specific interventions could policymakers implement to enhance adoption rates? The paper should elaborate on the role of agricultural extension services, access to credit, or other mechanisms that can facilitate the uptake of CSA practices.

 Although the study is methodologically sound, a clearer discussion of its limitations would enhance transparency. For example, were there any challenges in the data collection process, or potential biases in the selection of farmers? Additionally, suggesting avenues for future research, such as longitudinal studies on the long-term impacts of CSA adoption, would strengthen the study's contribution to the field.

Is the work clearly and accurately presented and does it cite the current literature?

Partly

If applicable, is the statistical analysis and its interpretation appropriate?

Partly

Are all the source data underlying the results available to ensure full reproducibility?

Yes

Is the study design appropriate and is the work technically sound?

Yes

Are the conclusions drawn adequately supported by the results?

Yes

Are sufficient details of methods and analysis provided to allow replication by others?

Yes

Reviewer Expertise:

livelihood vulnerability, climate change adaptation, extension methods

I confirm that I have read this submission and believe that I have an appropriate level of expertise to confirm that it is of an acceptable scientific standard, however I have significant reservations, as outlined above.

# Response

**Published:** 2024-10-26

**Authors:** chavula petros

**Affiliations:** Agricultural Economics, University of Zambia, Lusaka, Lusaka Province, Zambia

Dear Reviewers,

 I have thoroughly reviewed your previous comments and have addressed each one accordingly. I look forward to receiving any further feedback at your earliest convenience.

 Best regards,

# Reviewer report: Reviewer response for version 1

**DOI:** 10.5256/f1000research.158112.r305606

**Published:** 2024-08-10

**Authors:** Ibsa Dawid

**Affiliations:** 1 Oromia Agricultural Research Institute, Asella Agricultural Engineering Research Center, Asella, Ethiopia

1. Is the work clearly and accurately presented and does it cite the current literature? The work is not clearly accurate, not briefly presented and it’s bulky, not hit the target. Partly……, why because now we are in 2024, but the authors cited even ten years back reference, the climate change is the current issue so it’s appropriate if the author use the recent literatures not more than the last five years.

 2.  Is the study design appropriate and is the work technically sound?

 Partly, some steps are there but they are not fully designed appropriately.

 3. Are sufficient details of methods and analysis provided to allow replication by others?

 No, the methods and analysis provided are not sufficient.

 4.  ….is the statistical analysis and its interpretation appropriate?

 Not applicable. The statistical analysis is not applicable, for example a Cobb-Douglas production analysis……for what purpose the author used this analysis in the document? I did not seen it. The propensity sore matching is not well interpreted by with the steps. The formula for all the existing steps should be discussed.

 5. Are all the source data underlying the results available to ensure full reproducibility?

 No, the results presented do not seem to be fully consistent with the study's objectives, and they are not described in sufficient detail. The outcomes from Tables 10 and 11 are not clearly articulated, and there appear to be some inaccuracies in the model's results. It seems that the author may lack sufficient statistical experience, as there is difficulty in distinguishing which values are significant and which are not. The values in the tables are listed as they appear in the model, but it would be more effective if the results were first described, then compared with previous findings.

 Regarding Tables 10 and 11, while they seem to address the study's objectives, the results are not adequately explained. For instance, the values indicated in the tables may not align with established scientific standards. For example, in Table 11, the variable "household size" shows a t-value of 1.2800 and a p-value of 0.0012. It's unclear how these calculations were performed, as the conclusion drawn—that the variable significantly influenced farmers' crop productivity—does not seem accurate. Additionally, the interpretation of other variables may also require reconsideration.

 As a note, a variable is generally considered to have a significant influence if its t-value or z-value is greater than 1.65 at a 10% significance level.

 6. Are the conclusions drawn adequately supported by the results?

 No, the conclusions drawn do not seem to be fully supported by the study's results. For example, the authors conclude that all variables significantly influenced CSA practices and crop production. However, this does not align with the results presented in Table 10, where gender, farm size, livestock, and access to climate information did not significantly influence the adoption of CSA. Despite this, the authors concluded that these variables were significant and influenced adoption decisions.

 Similarly, in Table 10, the farm size shows a z-value of -0.4500 and a p-value of 0.0050, which raises questions about the conclusions drawn. A more robust justification would be helpful here. The same concern applies to the results in Table 11, where variables such as marital status, education, and household size were not shown to significantly influence crop productivity based on the results, which contradicts the conclusions made by the authors.

**Not Approved:** **“ **I have read this submission. I believe I have an appropriate level of expertise to state that I do not consider it to be of an acceptable scientific standard, for reasons outlined above.**”**

Is the work clearly and accurately presented and does it cite the current literature?

Partly

If applicable, is the statistical analysis and its interpretation appropriate?

Not applicable

Are all the source data underlying the results available to ensure full reproducibility?

No

Is the study design appropriate and is the work technically sound?

Partly

Are the conclusions drawn adequately supported by the results?

No

Are sufficient details of methods and analysis provided to allow replication by others?

No

Reviewer Expertise:

Climate-Smart Agriculture and Agricultural Economics

I confirm that I have read this submission and believe that I have an appropriate level of expertise to state that I do not consider it to be of an acceptable scientific standard, for reasons outlined above.
